# Does intraoperative fluoroscopy improve acetabular component positioning and limb-length discrepancy during direct anterior total hip arthroplasty? A meta-analysis

**DOI:** 10.1186/s13018-023-04023-w

**Published:** 2023-08-08

**Authors:** Changjiao Sun, Woo Guan Lee, Qi Ma, Xiaofei Zhang, Zhe Zhao, Xu Cai

**Affiliations:** 1 Department of Orthopedic, Beijing Tsinghua Changgung Hospital, School of Clinical Medicine, Tsinghua University, No.168 Litang Road, Dongxiaokou Town, Changping District, Beijing, 102218 China; 2 FRCS (Edinburgh) Kuching Specialist Hospital Sarawak, Tabuan Stutong Commercial Centre, 93350 Kuching Sarawak, Malaysia; 3 Department of Clinical Epidemiology and Biostatistics, Beijing Tsinghua Changgung Hospital, School of Clinical Medicine, Tsinghua University, No.168 Litang Road, Dongxiaokou Town, Changping District, Beijing, 102218 China

**Keywords:** Total hip arthroplasty, Intraoperative fluoroscopy, Component positioning, Limb-length discrepancy

## Abstract

**Background:**

The positioning of implant components for total hip arthroplasty (THA) is essential for joint stability, polyethylene liner wear, and range of motion. One potential benefit of the direct anterior approach (DAA) for THA is the ability to use intraoperative fluoroscopy for acetabular cup positioning and limb-length evaluation. Previous studies comparing intraoperative fluoroscopy with no fluoroscopy during DAA have reported conflicting results. This meta-analysis aimed to evaluate whether intraoperative fluoroscopy improves component positioning compared to no fluoroscopy during direct anterior total hip arthroplasty.

**Methods:**

A systematic review following the Preferred Reporting Items for Systematic Reviews and Meta-Analyses guidelines was conducted. We searched Web of Science, EMBASE, PubMed, Cochrane Controlled Trials Register, Cochrane Library, Highwire, CBM, CNKI, VIP, and Wanfang database in May 2023 to identify studies involving intraoperative fluoroscopy versus no fluoroscopy during direct anterior total hip arthroplasty. Finally, we identified 1262 hips assessed in seven studies.

**Results:**

There were no significant differences in terms of acetabular cup inclination angle (ACIA, *P* = 0.21), ACIA within safe zone rate (*P* = 0.97), acetabular cup anteversion angle (ACAA, *P* = 0.26); ACAA within safe zone rate (*P* = 0.07), combined safe zone rate (*P* = 0.33), and limb-length discrepancy (LLD, *P* = 0.21) between two groups.

**Conclusion:**

Even though intraoperative fluoroscopy was not related to an improvement in cup location or LDD. With fewer experienced surgeons, the benefit of intraoperative fluoroscopy might become more evident. More adequately powered and well-designed long-term follow-up studies were required to determine whether the application of the intraoperative fluoroscopy for direct anterior total hip arthroplasty will have clinical benefits and improve the survival of prostheses.

## Introduction

There is confusion and debate regarding the impact of intraoperative fluoroscopy on component position and limb-length discrepancy during direct anterior total hip arthroplasty (DATHA). According to certain studies [1–3], there was no statistically or clinically significant difference in acetabular inclination and anteversion or LLD between the groups who underwent fluoroscopy and those who did not. According to several other researches, intraoperative fluoroscopy during DATHA would enhance acetabular component location or limb-length disparity compared to no fluoroscopy [4, 5]**.** To our knowledge, no meta-analysis compares the use of intraoperative fluoroscopy and no intraoperative fluoroscopy during DATHA. So, we conducted a thorough systematic research analysis to evaluate the evidence comparing intraoperative fluoroscopy to no fluoroscopy during DATHA. Specifically, our goal was to compare the following: (1) acetabular cup inclination angle (ACIA); (2) ACIA within safe zone rate; (3) acetabular cup anteversion angle (ACAA); (4) ACAA within safe zone rate; (5) combined safe zone rate; and (6) limb-length discrepancy (LLD).

## Methods

The Preferred Reporting Items for Systematic Reviews and Meta-Analyses (PRISMA) statement’s requirements were followed for conducting the study [6]. This study’s protocol was made PROSPERO-registered (the International Prospective Register of Systematic Reviews), and the registration number was CRD42022316521.

### Search strategy

We conducted a literature screening for original articles published before May 1, 2023. We searched Web of Science, EMBASE, PubMed, Cochrane Controlled Trials Register, Cochrane Library, Highwire, CBM, CNKI, VIP, and Wanfang database to identify studies involving intraoperative fluoroscopy versus no fluoroscopy during direct anterior total hip arthroplasty. The keywords used were "total hip arthroplasty," "total hip replacement," "direct anterior approach," "fluoroscopy," "X-ray," manual in conjunction with Boolean operators, "AND" or "OR." We used the Review Manager software to perform the meta-analysis. Articles were preliminarily screened by two independent reviewers (W.G.L and Q.M.) using the title and abstract to identify those that met inclusion criteria. The full text of each study that passed a preliminary review was then subjected to full-text review by two reviewers (C.J.S and Z.Z.) using the same inclusion and exclusion criteria.

### Inclusion criteria

We identified and included all articles comparing intraoperative and no fluoroscopy during DATHA in the search strategy. If studies met the following requirements, they were included for further evaluation: (1) The THA procedure was performed with a direct anterior approach. (2) Intraoperative fluoroscopy was involved. (3) The comparator was no fluoroscopy in the comparative study. (4) One or more of the indices below were reported: ACIA, ACIA within safe zone rate, ACAA, ACAA within safe zone rate, combined safe zone rate, and LLD. We presented detailed definitions of some outcomes in Table 1. We excluded: (1) studies that revision of THA was performed. (2) Unclear or incomplete sample data were available.

**Table 1:** Definition of some outcomes

Outcome	Definition
ACIA	Viewed on a standard, weight-bearing AP radiograph, acetabular cup inclination is measured in degrees between a line drawn along the angle of the rim of the cup and the horizontal, trans-obturator foramen nadir reference line (a line drawn between the most inferior point of the obturator foramen)
ACAA	Acetabular anteversion was measured on the cross-table lateral image according to the method described by Woo and Morrey
LLD	Viewed on a standard, weight-bearing AP radiograph, LLD is measured in millimeters as the difference in perpendicular distance between the horizontal, trans-ischial reference line, and the medial tip of the lesser trochanter, as compared to the contralateral side. The trans-ischial line was chosen as reference as it has been validated in the literature as a reliable point of reference
FCOD	Viewed on a standard, weight-bearing AP radiograph, femoral offset difference is measured in millimeters as the difference in perpendicular distance between the longitudinal anatomic axis of the femur and the center of rotation of the femoral head, as compared to the contralateral side

ACIA, acetabular cup inclination angle; ACAA, acetabular cup anteversion angle; FCOD, femoral component offset difference; LLD, limb-length discrepancy. Definition of some outcomes including ACIA, ACAA, LLD, FCOD

### Data extraction process

The search strategy identified and included all articles comparing intraoperative and no fluoroscopy during direct anterior total hip arthroplasty. Two independent investigators screened each study for inclusion in the meta-analysis and independently extracted the data that were accessible from each study. We extracted the data based on the following: (1) research features (i.e., authors, year of publication, country, type of study), (2) population information (i.e., age, gender, body mass index (BMI), and follow-up time); and (3) clinical information (i.e., outcomes). If necessary results are omitted, we will email the authors to get further information.

### Data transformation

Some studies reported outcomes data using the median, minimum, and maximum values, or the median and first and third quartiles. We estimated the sample’s mean with the method presented by Luo et al. [7] and the sample’s standard deviation (SD) based on the method presented by Wan et al. [8] so that we could include these data in our meta-analysis. This method of estimating mean and standard deviation values has proven reliable [9–12].

### Assessment of studies

We used the nine-star Newcastle–Ottawa Scale (NOS), a proven, validated tool for evaluating the quality of non-randomized research, to rate the non-randomized studies’ methodological quality [13]. The NOS focused on the selection and comparability of cohorts and assessing outcomes and follow-up. Each study was evaluated for quality by two separate researchers, and a third researcher settled any disagreements.

### Statistical analysis

We performed all statistical analyses with Review Manager (version 5.4 for MAC, the Cochrane Collaboration, Copenhagen). Data were presented as mean ± SD. We used the *I*^2^ and Q test to evaluate the heterogeneity between studies. *P* values ≤ 0.1 or *I*^2^ value > 50% suggested high heterogeneity; thus, we used the randomized effects model. Otherwise, we used the fixed effects model [14]. The combined and individual effect sizes were estimated with 95% confidence intervals (CIs). In each study, we used the odds ratio (OR) and relevant 95% confidence interval (CI) to measure dichotomous variables such as ACIA within safe zone rate, ACAA within safe zone rate, and combined safe zone rate. Reported OR was supposed to approximate RR (relative risk) based on Cornfield’s rare disease outcome assumption [15]. We used the mean difference (MD) to assess continuous outcomes such as ACIA, ACAA, and LLD with a 95% confidence interval (CI). If the *P* values were less than 0.05, we regarded the results as having a statistically significant difference. The stability of the findings was evaluated using sensitivity analysis (if necessary).

## Results

### Search results

**Fig. 1:**
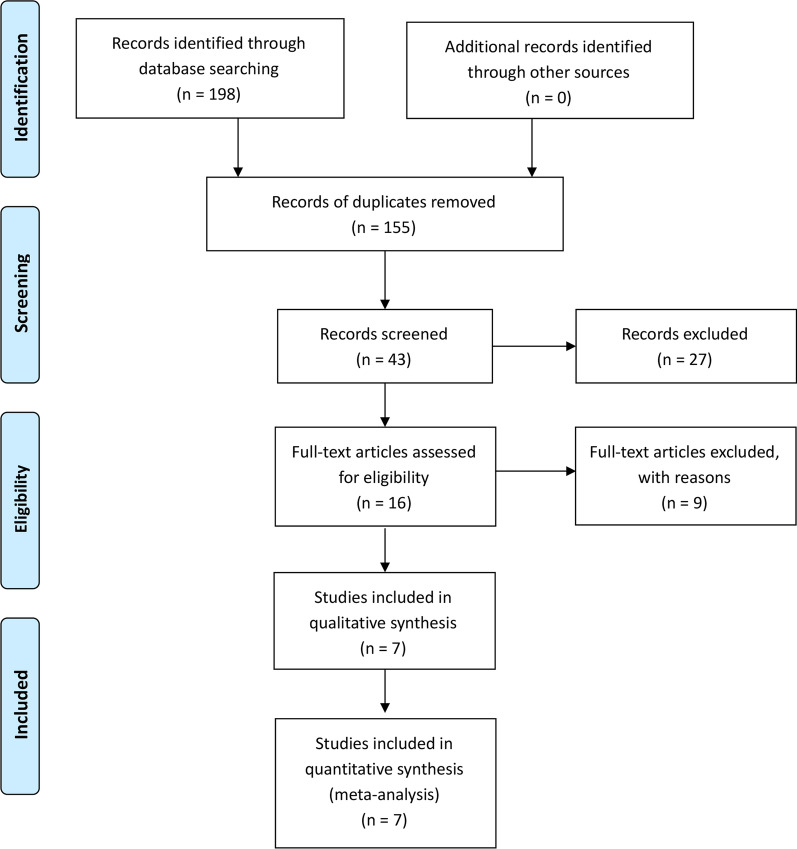
The literature search and selection process

Figure 1 depicts the literature search and selection process. Finally, seven publications were included in our meta-analysis. The PRISMA flow diagram in Fig. 1 shows the detailed literature screening process. According to the literature search strategy described earlier, 198 relevant citations were identified from the databases. After deleting 155 duplicates, we obtained 43 articles. Upon review of the titles and abstracts of the 43 articles, 27 irrelevant clinical studies were excluded. By reading the 16 full-text articles, we excluded another nine articles for the following reasons: systematic reviews, no compare groups, and no useful outcome data. The remaining seven articles were deemed appropriate. Finally, we identified 1262 patients (1262 THAs) assessed in seven articles.

### Study characteristics and quality

We presented detailed baseline characteristics information in Tables 2 and 3. All the included studies were published in English and Chinese between 2014 and 2021.

**Table 2:** The detailed baseline characteristics information

The detailed baseline characteristics information								
References	Country	Study type	Intraoperative fluoroscopy/no intraoperative fluoroscopy					
			Patients	THAs	Mean age (years)	Female gender (%)	BMI	Follow-up time(month)
Bingham [3]	USA	RCS	125/140	125/140	63.6/67.9	58/83	29.9/26.7	NA
Goodman [5]	USA	RCS	100/100	100/100	63.5/65.5	54/65	28.31/28.13	4/4
Holst [1]	USA	PCS	42/42	42/42	65.2/62.7	50/59.5	25.3/26	1.5/1.5
Hu [4]	China	RCS	50/50	50/50	57.5/63	50/50	22.5/22.9	6/6
Jennings [16]	USA	RCS	98/101	98/101	69/66	54.1/50.5	28.1/25.8	6/6
Leucht [17]	USA	RCS	100/100	100/100	59.3/60.3	52/57	28.3/28.9	1.5/1.5
Summers [2]	USA	RCS	154/60	154/60	59.4/52.7	NA	28.29/27.4	36/36

BMI, body mass index; THA, total hip arthroplasty; RCT, randomized control trial; RCS, retrospective cohort study; PCS, prospective cohort study. Summary of studies characteristics, including year of publication, country, study type, the number of patients, THAs, age, gender, BMI, and follow-up time of two groups. SE/$\sqrt{1/NE+1/NC}$

**Table 3:** The detailed information of outcomes

References	Outcome
Bingham [3]	ACIA, ACAA, LLD
Goodman [5]	ACIA, ACAA, ACIA within safe zone rate, ACAA within safe zone rate
Holst [1]	ACIA, ACAA, ACIA within safe zone rate, ACAA within safe zone rate, combined safe zone rate
Hu [4]	ACIA, ACAA, LLD
Jennings [16]	ACIA, ACAA, ACIA within safe zone rate, ACAA within safe zone rate, combined safe zone rate
Leucht [17]	ACIA, ACAA, LLD, ACIA within safe zone rate, ACAA within safe zone rate, combined safe zone rate
Summers [2]	ACIA, ACAA, ACIA within safe zone rate, ACAA within safe zone rate, combined safe zone rate

ACIA, acetabular cup inclination angle; ACAA, acetabular cup anteversion angle; FCOD, femoral component offset difference; LLD, limb-length discrepancy. The detailed information of outcomes in the included studies

### Risk-of-bias assessment

The included studies’ methodological quality scores ranged from seven to eight (Table 4). The overall quality of the studies that were included was therefore deemed adequate.

**Table 4:** Risk-of-bias assessment for the studies included in the meta-analysis (NOS)

Risk-of-bias assessment for the studies included in the meta-analysis (NOS)									
(nRCT) Study = 10	Selection				Comparability	Outcome/exposure			Score
	Item 1	Item 2	Item 3	Item 4	Item 5	Item 6	Item 7	Item 8	
Bingham [3]	*		*	*	**	*		*	7
Goodman [5]	*		*	*	**	*		*	7
Holst [1]	*		*	*	**	*		*	7
Hu [4]	*		*	*	**	*		*	7
Jennings [16]	*		*	*	**	*		*	7
Leucht [17]	*		*	*	**	*		*	7
Summers [2]	*		*	*	**	*	*	*	8

The methodological quality of the involved studies ranged from 7 to 8

Item 1, Is the case definition adequate/representativeness of the exposed cohort

Item 2, Representativeness of the case/selection of the non-exposed cohort

Item 3, Selection of controls/ascertainment of exposure to implants

Item 4, Definition of controls/demonstration that outcome of interest was not present at start of study

Item 5, Comparability of cases and controls on the basis of design or analysis/comparability of cohorts on the basis of the design or analysis

Item 6, Ascertainment of exposure/assessment of outcome

Item 7, Same method of ascertainment for cases and controls/was follow-up long enough for outcomes to occur

Item 8, Non-response rate/adequacy of follow-up of cohorts

### ACIA

**Fig. 2:**
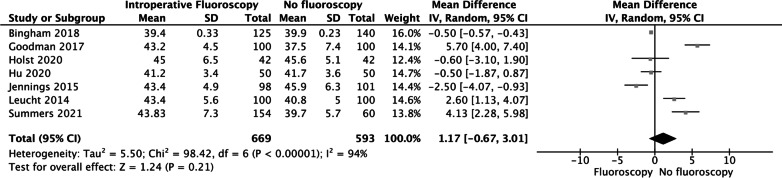
The pooled data showed that the ACIA was not significantly different between the two groups (MD = 1.17 95% CI [− 0.67, 3.01], *P* = 0.21)

Seven studies reported ACIA; the pooled data showed that the ACIA was not significantly different between the two groups (MD = 1.17 95% CI [− 0.67, 3.01], *P* = 0.21 Fig. 2).

### ACIA within safe zone rate

**Fig. 3:**
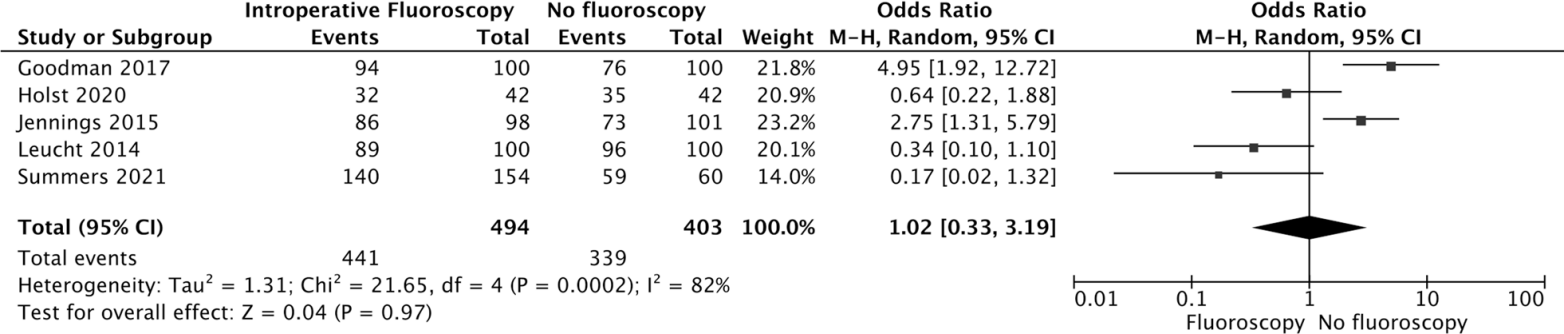
The forest plot revealed that both groups experienced similar ACIA rates (OR = 1.02, 95% CI [0.33, 3.19], *P* = 0.97)

Five studies reported the ACIA rate. The forest plot revealed that both groups experienced similar ACIA rates (OR = 1.02, 95% CI [0.33, 3.19], *P* = 0.97 Fig. 3).

### ACAA

**Fig. 4:**
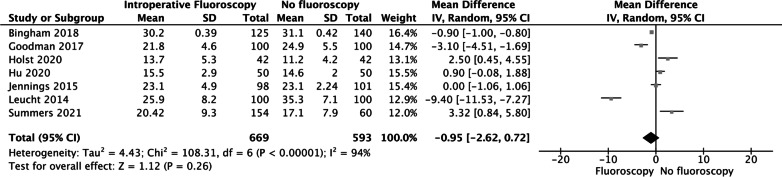
The forest plot revealed that both groups experienced similar ACAA (MD = -0.95, 95% CI [− 2.62, 0.72], *P* = 0.26)

Seven studies reported on the ACAA. The forest plot revealed that both groups experienced similar ACAA (MD = − 0.95, 95% CI [− 2.62, 0.72], *P* = 0.26 Fig. 4).

### ACAA within safe zone rate

**Fig. 5:**
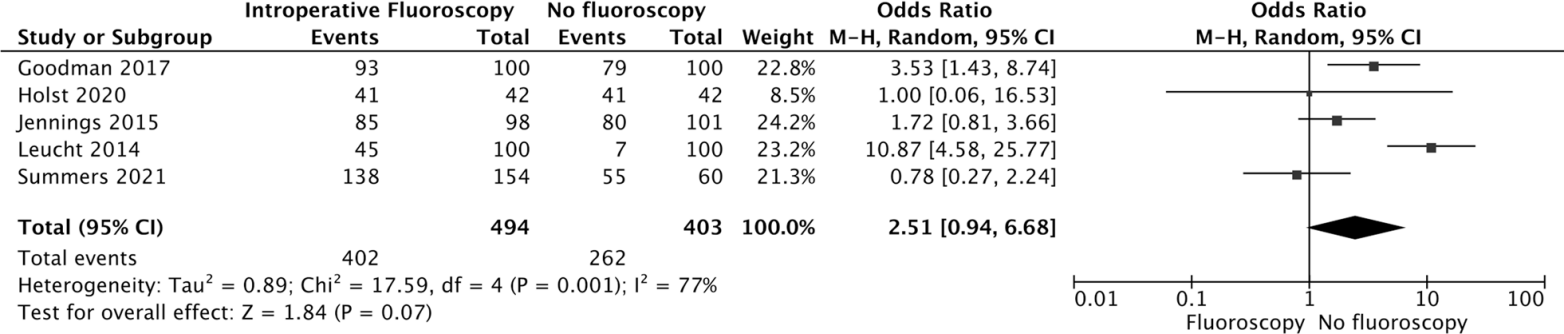
The forest plot revealed that both groups experienced similar ACAA rates (OR = 2.51, 95% CI [0.94, 6.68], *P* = 0.07)

Five studies reported the ACAA rate. The forest plot revealed that both groups experienced similar ACAA rates (OR = 2.51, 95% CI [0.94, 6.68], *P* = 0.07 Fig. 5).

### Combined safe zone rate

**Fig. 6:**
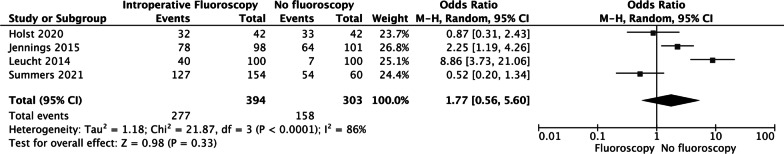
The forest plot revealed that both groups experienced similar combined safe zone rate (OR = 1.77, 95% CI [0.56, 5.60], *P* = 0.33)

Four studies reported a combined safe zone rate. The forest plot revealed that both groups experienced similar combined safe zone rate (OR = 1.77, 95% CI [0.56, 5.60], *P* = 0.33 Fig. 6).

### LDD

**Fig. 7:**
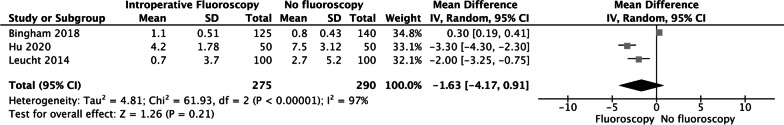
The forest plot revealed that both groups experienced similar LDD (MD = − 1.63, 95% CI [− 4.17, 0.91], *P* = 0.21)

Three studies reported LDD. The forest plot revealed that both groups experienced similar LDD (MD = − 1.63, 95% CI [− 4.17,0.91], *P* = 0.21 Fig. 7).

## Discussion

The study’s key findings include the lack of a statistically significant difference between the two groups regarding cup anteversion and inclination measurements. Additionally, there were no discernible variations in the detection of LLD between the two groups.

One of the most crucial elements in the success of THA is the proper location of the acetabular component. Harrison et al. [18] and Lewinnek et al. [19] both reported on the impact of cup abduction and anteversion on the chance of dislocation. The distance that the femoral head must travel to dislocate is shortened when the cup is positioned too vertically, anteverted, or retroverted [18, 19, 19, 20]. Lewinnek suggested a "safe zone" of 30° to 50° of abduction and 5° to 25° of anteversion for the insertion of acetabular components [19]. Additionally, longer-term findings demonstrate that cup position outside the safe zone range has been linked to decreased bone support, higher polyethylene wear, edge loading, impingement, ceramic squeaking, and increased rates of adverse tissue reaction in metal-on-metal hips.

Numerous methods have been developed to optimize component placing, including using anatomic landmarks, intraoperative radiographs, and more modern technology such as computer navigation, robotics, computer navigation, and patient-specific positioning devices [21–25]. Fluoroscopy is frequently employed to achieve the appropriate anteversion and inclination of the acetabular component [29, 32].

Recent years have seen an upsurge in using the direct anterior approach (DAA) for THA [26]. The approach is said to have a variety of advantages, according to its proponents, including a slight advantage in early recovery [27], a low dislocation rate [28], and excellent radiographic component placement parameters [29]. The DAA’s ability to capture intraoperative fluoroscopic pictures while the patient is supine for implant placement is another advantage [30, 31]. Some surgeons have highlighted fluoroscopy’s simplicity of use as a potential advantage of the strategy. It might increase surgical accuracy for acetabular component location and determining leg length, enhancing wear rates, range of motion, and stability. However, our findings showed that intraoperative fluoroscopy did not significantly improve implant location and leg-length assessment during DATHA. The results of direct anterior total hip arthroplasty with fluoroscopy are comparable to those without fluoroscopy.

When assessing the results of our meta-analysis, there are additional considerations to make. Most of the data used in the current meta-analysis came from hospitals where the surgeons were skilled in doing DATHA. Generally, the surgeon’s training level conducting DATHA affects the likelihood of problems [32]. Although the included studies did not discuss the benefits of fluoroscopy for surgeons with less experience or surgeons in lower-volume hospitals, this group of surgeons is expected to benefit more from intraoperative fluoroscopy. However, there are also potential disadvantages related to its use, including the extra time required to get the images, higher costs, radiation exposure for both the patient and surgical team, and some worry that the sterile fluoroscopy arm covering may become contaminated during the operation [33–38]. If the patient benefits from these drawbacks, these disadvantages may be acceptable.

Although many surgeons have used the so-called safe zone as their paradigm, recent research has called into question this idea [39], with the revelation that dislocation is more complex than simply taking into account acetabular component angulation characteristics [40]. Because cementless components are comprised of materials that have different levels of radio-opacity, measuring anteversion with intraoperative fluoroscopy can be difficult and inaccurate. Particularly in these situations, determining the proper posture could be best guided by markers from the local anatomy. We should also consider the acetabular cup’s orientation to the specific patient conditions, including hip-spine pathology, spinal stiffness, or a defective anterior wall [41].

It is important to keep in mind the limitations of the data set while evaluating our results. Firstly, there is a paucity of prospective, comparative studies and randomized controlled trials, which may have reduced the quality of the evidence for this meta-analysis. The results and conclusions need to be confirmed by other prospective randomized trials examining additional clinical indicators, even though we have already included all relevant studies and made an effort to gather more data for this meta-analysis and to evaluate its impact. Secondly, there was an essential variability between the studies with respect to the different variations in the radiographs obtained. Obtaining pelvis radiographs is standardized to center the pubic symphysis over the coccyx and to obtain them standing with a marker ball. However, despite this, there could be slight differences in rotation between radiographs, causing some variability in the radiographic measurements. Thirdly, these studies’ follow-up duration is still short. Studies with longer follow-ups and well-defined groups randomized to DATHA with or without an intraoperative radiograph would provide valuable data for analysis. Fourthly, our meta-analysis purely discusses radiographs findings (inclination and anti-version angles, as well as LLD). We do not analyze the dislocation rate. Because there are many factors associated with dislocation, the position of the prosthesis on imaging is only one of the influencing factors. Despite these limitations, the meta-analysis used the right approach and included some papers that provided information on numerous measurement outcomes from the intraoperative fluoroscopy and no fluoroscopy groups.

## Conclusion

Even though intraoperative fluoroscopy was not related to an improvement in cup location or leg-length discrepancy, it should be emphasized that with fewer experienced surgeons, the benefit of intraoperative fluoroscopy might become more obvious. More adequately powered and well-designed long-term follow-up studies were required to determine whether the application of the intraoperative fluoroscopy for direct anterior total hip arthroplasty will have clinical benefits and improve the survival of prostheses.

## Data Availability

The data sets generated during and/or analyzed during the current study are available from the corresponding author on reasonable request.

## Abbreviations

THA: Total hip arthroplasty
DAA: Direct anterior approach
DATHA: Direct anterior total hip arthroplasty
ACIA: Acetabular cup inclination angle
ACAA: Acetabular cup anteversion angle
LLD: Limb-length discrepancy
CIs: Confidence intervals
RR: Risk ratio
OR: Odds ratio
VMD: Weighted mean difference
BMI: Body mass index
RCTs: Randomized controlled trials
RCS: Retrospective cohort study
PCS: Prospective cohort study
EMBASE: Excerpta Medica Database
CENTRAL: Cochrane Central Register of Controlled Trials
CNKI: China National Knowledge Infrastructure
